# Retraction: MicroRNA-216a inhibits the metastasis of gastric cancer cells by targeting JAK2/STAT3-mediated EMT process

**DOI:** 10.18632/oncotarget.28702

**Published:** 2025-03-10

**Authors:** Youmao Tao, Songbai Yang, Yuanyu Wu, Xuedong Fang, Yannan Wang, Yan Song, Tao Han

**Affiliations:** ^1^Department of Gastrointestinal Colorectal and Anal Surgery, China-Japan Union Hospital of Jilin University, Changchun, Jilin Province 130033, China; ^2^Department of Vascular Surgery, China-Japan Union Hospital of Jilin University, Changchun, Jilin Province 130033, China

**This article has been retracted:** Oncotarget has completed its investigation of this paper, where it was verified that several figure images were duplicated from previously published papers. In particular, the wound healing assay images In Figure 2 (panels B and E) and Figures 7B and 8B are duplicates of wound healing assay images from four different earlier papers [1–4]. Transwell assay images in Figure 2, panels C and F, and in Figures 7C and 8C were found to be duplicates of transwell assay images in another earlier article that has since been retracted [5]. Figures 4A, 5C, 6 and 7A duplicate numerous western blot images from unrelated papers [6–8], one of which has since been retracted [8]. And Figure 6 has reused western blot images from Figure 5C and from earlier paper from the same lab [9]. The authors have been unresponsive in our attempts to contact them for more information regarding these duplications. As a result, the Editorial decision was made to retract this paper.

Original article: Oncotarget. 2017; 8:88870–88881. 88870-88881. https://doi.org/10.18632/oncotarget.21488
